# Comprehensive analysis of the UDP-glucuronate decarboxylase (UXS) gene family in tobacco and functional characterization of *NtUXS16* in Golgi apparatus in *Arabidopsis*

**DOI:** 10.1186/s12870-023-04575-3

**Published:** 2023-11-08

**Authors:** Zhimin Li, Runping Chen, Yufang Wen, Hanxiang Liu, Yangyang Chen, Xiaoyu Wu, Youxin Yang, Xinru Wu, Yong Zhou, Jianping Liu

**Affiliations:** 1 Jiangxi Engineering Laboratory for the Development and Utilization of Agricultural Microbial Resources, College of Bioscience and Bioengineering, Jiangxi Agricultural University, Nanchang, China; 2 College of Bioscience and Bioengineering, Jiangxi Agricultural University, Nanchang, China; 3 Jiangxi Key Laboratory for Postharvest Technology and Nondestructive Testing of Fruits & Vegetables, Collaborative Innovation Center of Post-Harvest Key Technology and Quality Safety of Fruits and Vegetables, College of Agronomy, Jiangxi Agricultural University, Nanchang, China; 4 Tobacco Research Institute, Chinese Academy of Agricultural Sciences, Qingdao, China; 5 Key Laboratory of Crop Physiology, Ecology and Genetic Breeding, Ministry of Education, Jiangxi Agricultural University, Nanchang, China

**Keywords:** UDP-glucuronate decarboxylase, Golgi apparatus, Tobacco, Hypocotyl length, Root length

## Abstract

**Background:**

UDP-glucuronate decarboxylase (also named UXS) converts UDP-glucuronic acid (UDP-GlcA) to UDP-xylose (UDP-Xyl) by decarboxylation of the C6-carboxylic acid of glucuronic acid. UDP-Xyl is an important sugar donor that is required for the synthesis of plant cell wall polysaccharides.

**Results:**

In this study, we first carried out the genome-wide identification of *NtUXS* genes in tobacco. A total of 17 *NtUXS *genes were identified, which could be divided into two groups (Group I and II), and the Group II UXSs can be further divided into two subgroups (Group IIa and IIb). Furthermore, the protein structures, intrachromosomal distributions and gene structures were thoroughly analyzed. To experimentally verify the subcellular localization of NtUXS16 protein, we transformed tobacco BY-2 cells with NtUXS16 fused to the monomeric red fluorescence protein (mRFP) at the C terminus under the control of the cauliflower mosaic virus (CaMV) 35S promoter. The fluorescent signals of NtUXS16-mRFP were localized to the medial-Golgi apparatus. Contrary to previous predictions, protease digestion analysis revealed that NtUXS16 is not a type II membrane protein. Overexpression of *NtUXS16* in Arabidopsis seedling in darkness led to a significant increase in hypocotyl length and a reduction in root length compared with the wild type. In summary, these results suggest Golgi apparatus localized-NtUXS16 plays an important role in hypocotyl and root growth in the dark.

**Conclusion:**

Our findings facilitate our understanding of the novel functions of *NtUXS16* and provide insights for further exploration of the biological roles of *NtUXS* genes in tobacco.

**Supplementary Information:**

The online version contains supplementary material available at 10.1186/s12870-023-04575-3.

## Introduction

UDP-xylose (UDP-Xyl) plays a crucial role in plants as a key sugar donor, essential for the synthesis of various plant cell wall polysaccharides, plant metabolites, and glycoproteins [1–5]. UDP-Xyl is synthesized through a two-step biochemical conversion process catalyzed by UDP-glucose dehydrogenase (UGD) and UDP-glucuronate decarboxylase, also known as UXS. This conversion involves the transformation of UDP-glucose (UDP-Glc) into UDP-Xyl [6]. In the initial step, the cytosolic enzyme UGD facilitates the conversion of UDP-Glc to UDP-GlcA while simultaneously producing two molecules of NADH through the reduction of NAD^**+**^ [7, 8]. Subsequently, in the second step, UXS performs the decarboxylation of the C6-carboxylic acid of glucuronic acid, resulting in the conversion of UDP-GlcA to UDP-Xyl [9].

Since the initial discovery of the UDP-GlcA decarboxylase cDNA in the fungus *Cryptococcus neoformans*, numerous biosynthetic genes encoding UXS have been successfully identified and isolated from Arabidopsis [10, 11], tobacco [12], pea [13], and rice [14–16]. In *Arabidopsis thaliana*, there are six UXS isoforms classified into two types: membrane proteins (AtUXS1, AtUXS2, and AtUXS4) and cytoplasm proteins (AtUXS3, AtUXS5 and AtUXS6) [11]. Cytosol-localized UXS, predominantly expressed in secondary wall-forming cells, plays a dominant and crucial role in the biosynthesis of xylan, in contrast to the Golgi-localized UXS [11, 17, 18]. However, the function of Golgi-localized UXSs is still not fully understood.

In tobacco, altering the expression of NtUXS through sense and antisense down-regulation resulted in significant changes to the hemicellulose content of the cell wall, pulping properties, and overall cell wall composition [19]. NtUXS16 shows limited similarity to the other N terminus amino acid sequences. It also has a single transmembrane domain, similar to AtUXS1 and AtUXS2, suggesting that NtUXS16 may be a type II membrane protein located in the Golgi [11, 20]. However, the physiological roles of membrane-localized UXS in tobacco are not well understood. Therefore, this study aims to identify and characterize the UXS gene family in the tobacco genome, analyze the evolutionary relationships among NtUXS family members, confirm the localization and membrane topology, and investigate the function of NtUXS16 in Arabidopsis.

## Materials and methods

### Identification and characterization of the *UXS* gene family in tobacco genome

To identify the *UXS* gene family members in cultivated tobacco (*Nicotiana tabacum*), all the downloaded AtUXS and OsUXS protein sequences from TAIR (The Arabidopsis Information Resource, http://www.arabidopsis.org/) and Phytozome (https://phytozome-next.jgi.doe.gov/) were used as queries for BLASTP search against tobacco genome database from China tobacco genome database V2.0 “tobacco genome sequencing project” (http://www.tobaccodb.org/). The integrity for the conserved UXS domain organizations was checked through Pfam (https://pfam.xfam.org/) and SMART (http://smart.embl-heidelberg.de/). Using the tools provided by ProtParam (http://web.expasy.org/protparam/), theoretical molecular weight (MW), isoelectric point (pI), and grand average of hydropathicity (GRAVY) of the NtUXS proteins were estimated. The transmembrane domains of each NtUXS protein were predicted using TMHMM Server version 2.0 (https://services.healthtech.dtu.dk/service.php?TMHMM-2.0).

### Phylogenetic relationship, conserved motif and exon–intron structure analyses of *UXS* gene family in tobacco

To study the phylogeny of the *UXS* genes in tobacco, the full-length UXS protein sequences from *Nicotiana tabacum*, *Arabidopsis thaliana*, *Populus tomentosa*, and *Oryza sativa* were aligned by Clustal Omega (https://www.ebi.ac.uk/Tools/msa/clustalo/) with default parameter settings, and the MEGA7.0 software was used to create the phylogenetic tree of the UXS proteins using the NJ (neighbor-joining) method via bootstrap analysis with 1000 replicates. We use the online MEME website (http://meme-suite.org/) tool to determine the conserved motifs of NtUXS proteins. The maximum number of motifs was 10 and other parameter settings were set as default, and then the 10 identified motifs were illustrated with TBtools software [21]. The exon–intron structure of each *NtUXS* gene was visualized using the GSDS server (Gene Structure Display Server, http://gsds.gao-lab.org/).

### Isolation of *NtUXS16* cDNA

A tobacco full-length enriched cDNA library was constructed from an mRNA fraction prepared from *BY-2* cells treated with jasmonate into pGCAPsp2 vector as described previously [22, 23]. To assess the quality of library, the 5’ sequences of 609 clones were analyzed and the encoded proteins were predicted by a BLASTx search. Among them, *NtUXS16* were isolated from this library by PCR.

### Cell culture, construction of plasmid and transformation into BY-2 cell

The tobacco BY-2 cell cultures were carried out as described previously [24]. Log phase cells (3-day-old cell culture after subculture) were used throughout the study. To construct NtUXS16 fused with mRFP (monomeric red-fluorescent protein), the cDNA encoding tobacco NtUXS16 was amplified by PCR using a primer that contained Hind III and Xba I in the Hind III-Xba I sites of a derivative of the binary vector pMAT137, containing an mRFP-coding sequence for NtUXS16-mRFP. This construct was used for the *Agrobacterium*–mediated transformation of BY-2 cells [25].

### Treatment of tobacco cells with brefeldin A (BFA)

BFA (5 µg/ml; 17.8 mM final concentration) was added to the transformed tobacco cells and then incubated for 2 h. Control treatments were performed with an equal amount of DMSO.

### SDS-PAGE and detection of fluorescent protein

The protein sample was mixed with SDS sample buffer and applied to wells containing 12.5% polyacrylamide gel without heating. After electrophoresis, the gel was scanned by Typhoon 9400 scanner (GE Healthcare Bio-science, Boston, MA) to detect green and red fluorescent proteins. Image Quant software version 5.0 was used to analyze the images.

### Fluorescence confocal microscopy

For confocal microscopic analysis, we utilized an OLYMPUS IX80 fluorescence microscope (Olympus, Tokyo, Japan) equipped with a DSU confocal unit and an Uplan SApo 60 × /0.90 lens. GFP and RFP excitation/emission filter sets (Olympus) were employed. The obtained images were processed and analyzed using MetaMorph software (Molecular Devices, Sunnyvale, CA). To observe living tobacco BY-2 cells, they were mounted on glass slides following the previously described method [24].

### Antibodies and immunoblot analysis

An antibody against NtUXS16 was generated by immunizing rabbits with the synthetic peptide CYTPKPRKPWQNVIRP. The peptide synthesis and antibody production were conducted by GL Biochem in Shanghai, China. For western blot analysis, specific affinity-purified antibodies were used at a concentration of 0.48 µg/ml. The western blotting utilized anti-RFP (MBL; 1:1000) or anti-GFP (MBL; 1:2500) antibodies. Immunofluorescent detection was performed using polyclonal Alexa fluor-568 anti-rabbit secondary antibody or Alexa fluor-555 goat anti-mouse secondary antibody. Fluorescent signals on the PVDF membrane were detected using the Typhoon 8600 image analyzer from Molecular Dynamics Inc.

### Preparation of microsomal fraction

The microsomal fraction of BY-2 cells was prepared following the method described by Liu et al. (2015). Briefly, 20 g of BY-2 cells were harvested through filtration and subsequently homogenized in an extract buffer containing 0.45 M sucrose, 2 mM DTT, 5 mM Tris-MES (pH 7.3), and 2 mM MgCl_2_. The homogenate was then subjected to centrifugation at 1,000 g for 10 min at 4 ºC, repeated three times, followed by another centrifugation at 10,000 g for 10 min at 4 ºC. The resulting supernatant was carefully layered on top of a solution composed of 15 ml of 45% (w/v) sucrose and 15 ml of 20% (w/v) sucrose. Subsequently, the mixture was centrifuged at 100,000 g for 1 h. The middle layer in the tube was collected and utilized as the microsomal fraction.

### Protease digestion assay

In order to examine the impact of trypsin and/or Triton X-100 on microsomal fractions, we subjected the samples to treatment with or without these agents. To inhibit any additional proteolytic activity, we supplemented the treated samples with 10 mg/ml soybean trypsin inhibitor. The resulting digested mixtures were then separated using SDS-PAGE and immunoblot.

### Generation of Arabidopsis transgenic plants

For the generation of NtUXS16-overexpression transgenic plants, a new expression vector was generated. The CDS of NtUXS16 was amplified from construct pNL105 with primer pairs listed in Supplemental Table S1, and inserted into pCHF3 binary vector harboring the 35S promoter after digestion with *Pst* I and *Xba* I sites of the vector to produce the vector Pro35S:NtUXS16. The vector was introduced into *Agrobecterium tumefaciens* strain GV3101 and then into Arabidopsis (Columbia ecotype) plants using the *Agrobecterium*-mediated floral-dip method [26]. Transgenic T_1_ generation was selected on MS media containing 50 mg L^−1^ kanamycin (Catalog 25389–94-0, Sangon Biotech, China). Two positive lines in the T_2_ generation, exhibiting a 3:1 segregation ratio for kanamycin resistant sensitive, were selected to generate homozygous transgenic plants (T_3_) for further analysis. The phenotype of the T_3_ transgenic lines and wild-type Columbia plants were analyzed at maturation.

### RNA extraction and qRT-PCR analyses

Real-time quantitative PCR (qRT-PCR) was performed to analyze the expression of NtUXS16. Total RNA was extracted from 4-week-old plants using the Trizol (Invitrogen) according to the manufacturer’s instructions. 1 μg of total RNA was used in a reverse transcription reaction with the HiScript® II 1 st Strand cDNA Synthesis Kit (+ gDNA wiper) (Vazyme, China). cDNA was used as a template for qRT-PCR and diluted to volumes equivalent to 10 ng of total RNA μl^−1^ with nuclease-free water. qRT-PCR analysis of the targets was perform using the ChamQ SYBR qPCR Master Mix **(**Vazyme, China**)** with primer pairs listed in Supplemental Table S1. The qRT-PCR reactions were conducted with the following protocol: 95 ℃ for 30 s, followed by 40 cycles of 95 ℃ for 10 s, 60 ℃ for 30 s, 95 ℃ for 15 s, 60 ℃ for 60 s, and 95 ℃ for 15 s. The PCR reactions, cycling protocol and melting curves were performed according to the manufacturer's instructions. Each sample was represented by three biological and two technical repeats. *Actin8* was used to normalize the expression levels of the target genes, which were analyzed using the relative quantification method ΔΔCt. Statistical significance was reported by the Student's *t*-test.

### Plant materials and growth conditions

*Arabidopsis thaliana* (Col-0) was used in this study. For seedlings grown on an agar plate, seeds were surface-sterilized using 75% ethanol (v/v) for 3 min, and then washed twice with 90% ethanol (v/v) for 1 min, and then washed with ddH_**2**_O three times. The sterilized seeds were placed on a 1/2 MS with 0.8% plant agar plate. After being incubated at 4 °C for 72 h, seed plates were placed in a growth chamber for germination. Cultivated in growth chamber which was set at 20–22 °C with relative humidity of 60–70% under long-day conditions (16 h light/8 h dark), the light intensity was 110 μmol photons m^−2^ s^−1^ white light.

### Hypocotyl length quantification

Seeds were spread on 1/2 MS 0.8% agarose plant medium. After 3 d of vernalization, the seedlings were normally cultured in light incubator for 24 h to germinate. The seedlings were grown in the dark for 5 d and photographed with Epson Perfection V370 Photo. Import the picture into the ImageJ software (https://imagej.nih.gov/ij/download.html.) for measurement and analysis. Length is measured by converting into millimeters using an internal standard. Use the segmented line tool to trace along the length of the hypocotyl, generating a measurement and the value table by ImageJ [27]. At least 30 individuals were measured for each data point. Error bars refer to the SE.

## Results

### Genome-wide identification and sequence analyses of tobacco UXS genes

A total of 17 UXS genes in tobacco were identified by comprehensive analysis in tobacco genome dataset using BLASTP, and they were named as NtUXS1–NtUXS17, based on their phylogenetic positions (Table 1). The gDNA lengths of the NtUXS genes ranged from 2497 bp (NtUXS11) to 6879 bp (NtUXS13), with CDS lengths ranged from 972 bp (NtUXS5) to 1341 bp (NtUXS16), and these NtUXS genes encoded polypeptides ranged from 323 amino acids (NtUXS5) to 446 amino acids (NtUXS16) (Table 1). The theoretical MWs of these predicted NtUXS proteins ranged from 36.31 kDa to 49.85 kDa, and the pIs ranged from 5.69 to 9.61 (Table 1). Notably, the GRAVY values of NtUXS proteins were ranged from -0.408 to -0.218, indicating that these proteins were hydrophilic. In addition, transmembrane prediction of NtUXS proteins by TMHMM tool showed that 10 NtUXSs (NtUXS1, NtUXS2, NtUXS4, NtUXS7, NtUXS10, NtUXS13–17) have a transmembrane domain at their N-terminus (Figure S1). To further determine the sequence features of the 17 NtUXS proteins, their amino acid sequences were aligned, and the results revealed that all NtUXS proteins contained conserved motifs GxxGxxG and YxxxK (Figure S2).

**Table 1:** Identification and characterization of UXS gene family in *Nicotiana tabacum*

Gene	Chromosome	Chromosomal position	gDNA (bp)	CDS length (bp)	Protein physicochemical characteristics			
					**Length** **(aa)**	**MW (KDa)**	**pI**	**GRAVY**
*NtUXS1*	Chr02	59322211–59327651	5441	1284	427	47.74	8.30	-0.278
*NtUXS2*	Chr03	199877027–199883545	6519	1227	408	45.95	8.22	-0.218
*NtUXS3*	Chr05	135579414–135582216	2803	1068	355	39.95	5.95	-0.341
*NtUXS4*	Chr06	118412480–118416738	4259	1269	422	47.26	8.58	-0.273
*NtUXS5*	Chr07	15099331–15105701	6371	972	323	36.31	5.69	-0.290
*NtUXS6*	Chr08	9089391–9093914	4524	1041	346	39.02	8.59	-0.379
*NtUXS7*	Chr10	58477–60993	2517	1188	395	43.93	9.61	-0.307
*NtUXS8*	Chr15	14757611–14760943	3333	1032	343	38.73	7.10	-0.408
*NtUXS9*	Chr16	70238594–70241955	3362	1032	343	38.73	7.10	-0.406
*NtUXS10*	Chr19	52389910–52396403	6494	1206	401	44.82	8.61	-0.300
*NtUXS11*	Chr20	11209930–11212426	2497	1032	343	38.67	6.41	-0.387
*NtUXS12*	Chr21	61066815–61071004	4190	1041	346	39.09	8.28	-0.402
*NtUXS13*	Chr23	19020040–19026918	6879	1296	431	48.56	9.13	-0.354
*NtUXS14*	Chr24	2223930–2228579	4650	1278	425	48.04	8.83	-0.259
*NtUXS15*	Ntscaffold_1091	73043–79669	6627	1278	425	47.96	8.48	-0.258
*NtUXS16*	Ntscaffold_274	2661,387–2663991	2605	1341	446	49.85	9.37	-0.301
*NtUXS17*	Ntscaffold_317	2094461–2097065	2605	1251	416	46.31	9.55	-0.295

### Phylogenetic analysis of NtUXS family members

**Fig. 1:**
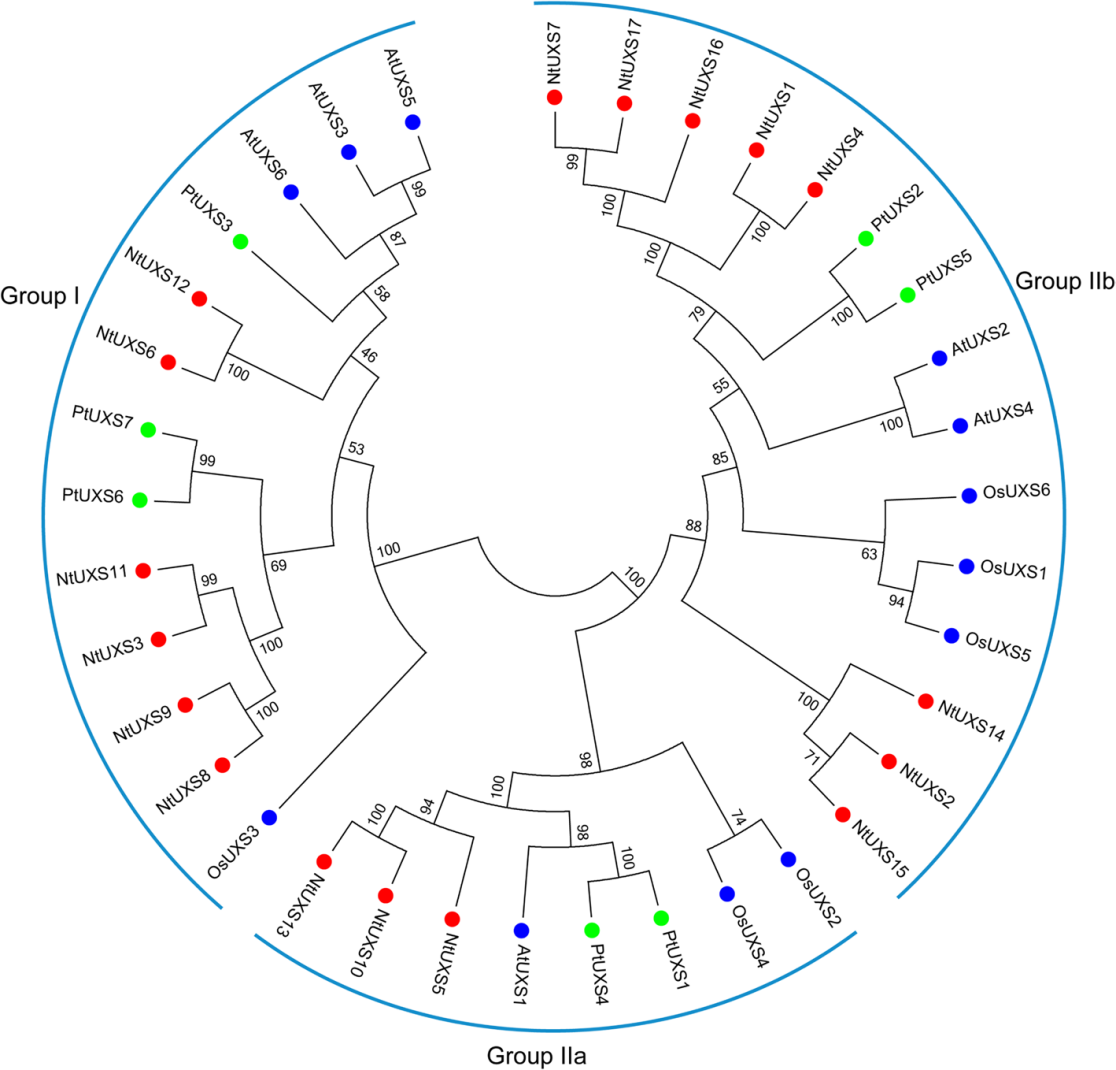
The phylogenetic relationship of the UXS gene family in *Nicotiana tabacum*, *Arabidopsis thaliana*, *Populus tomentosa*, and *Oryza sativa*. The IDs of UXS family genes used for phylogenetic relationship analysis are listed in Table S2

To study the evolutionary relationships of NtUXS family members, a phylogenetic tree was generated based on the full-length protein sequences of UXSs from *Nicotiana tabacum*, *Arabidopsis thaliana*, *Populus tomentosa*, and *Oryza sativa* using the NJ method. The result revealed that these UXSs can be divided into two groups (Group I and II), and the Group II UXSs can be further divided into two subgroups (Group IIa and IIb) (Fig. 1). Among them, NtUXS3, NtUXS6, NtUXS8, NtUXS9, NtUXS11, and NtUXS12 were devoid of transmembrane helices and clustered in Group I. NtUXS5, NtUXS10, and NtUXS13 were classified under Group IIa, while other NtUXSs were grouped in Group IIb (Fig. 1).

### Conservative motif distribution and exon–intron structure of tobacco UXS gene family

**Fig. 2:**
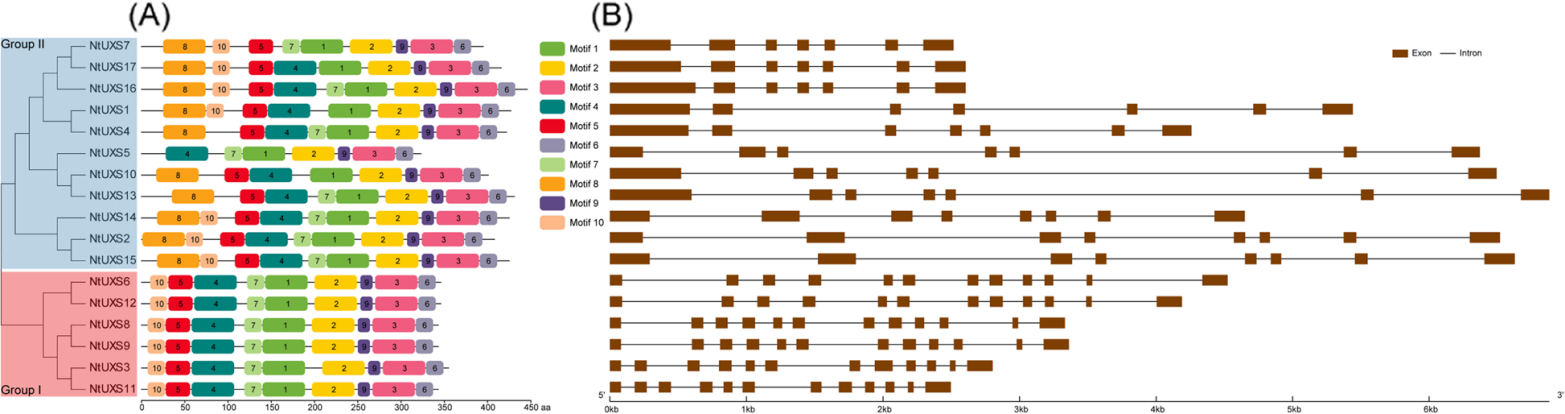
The conserved motif and gene structure analyses of tobacco *UXS* genes according to the phylogenetic relationship. The left part shows the phylogenetic relationships of 17 NtUXS proteins. **A** Conserved motif distribution of NtUXS proteins. The different colored boxes represent the conserved motifs, and the details of the motifs are presented in Table S3. **B** Gene structures of tobacco *UXS* genes. The brown boxes and black lines represent exons and introns, respectively

To study the diversity of NtUXS proteins, we examined the conserved motifs within them using the MEME program. As a result, a total of 10 motifs were identified, and the motifs were generally well conserved. For example, members in Group I shared conserved motif distributions, and all of them lacked motif 8. Most members in Group II contained all the 10 motifs, except for NtUXS7 (lack motif 4), NtUXS1 and NtUXS7 (lack motif 7), NtUXS5 (lack motifs 5, 8 and 10), NtUXS4 and NtUXS13 (lack motif 10), NtUXS10 (lack motifs 7 and 10) (Fig. 2A). The conservative motif distribution supports the results of the phylogenetic analysis.

The exon–intron structures of the NtUXS genes were determined according to their coding sequences and corresponding genomic sequences. Structural analysis revealed that the length, arrangement, and position of introns in NtUXS genes were quite conserved. And the intron numbers in members of Group I and II were 11 and 6, respectively (Fig. 2B).

### Cloning and subcellular localization of NtUXS16

**Fig. 3:**
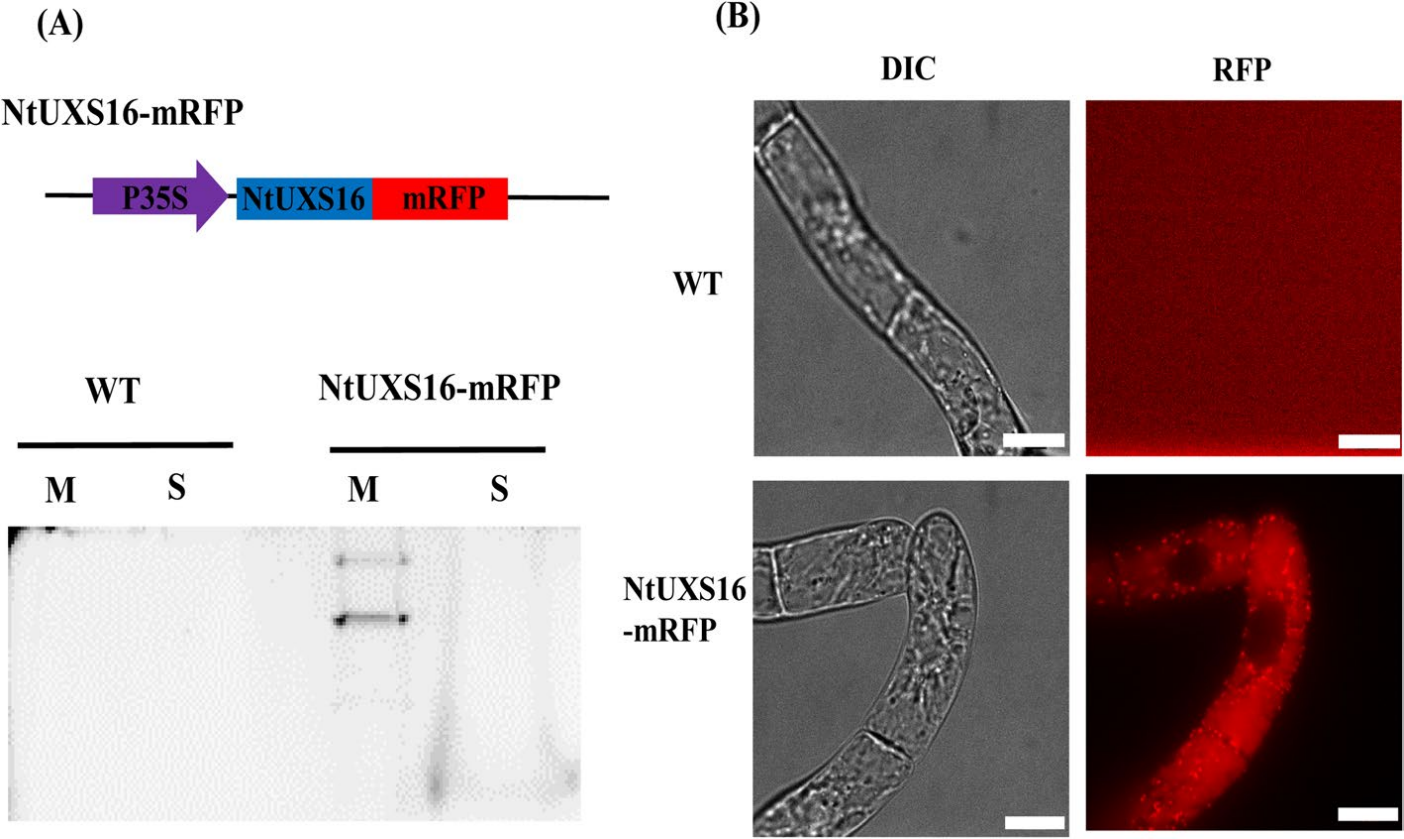
**A** NtUXS16-mRFP fusion protein was constructed under the of the CaMV 35S Promoter and fused with mRFP at C-terminal. The membrane and soluble fraction were prepared from non-transformed and transformed BY-2 cells by ultracentrifugation. After resolution of the proteins of sodium dodecyl sulfate (SDS)-polyacylamide gel electrophoresis (PAGE), only proteins in the membrane fraction from transgenic cell gave a fluorescent band at 68 kDa and another band (**B**) Localization of NtUXS16-mRFP fusion protein in BY-2 cells. Wild type, non-transformed cell was control in the same condition of detecting the localization of NtUXS16-mRFP fusion protein. NtUXS16-mRFP showed a punctate pattern of red fluorescence at the log phase (3-day old cell after subcultured), Bars = 10 µm

NtUXS16 was isolated from a tobacco full-length enriched cDNA library by PCR, which was constructed from an mRNA fraction prepared from BY-2 cells treated with jasmonate into pGCAPsp2 vector. NtUXS16 encoded a putative protein of 446 amino acids with a predicted transmembrane domain (TMD) in its N-terminal region (Figure S1). To analyze the localization of NtUXS16 in tobacco BY-2 cells, NtUXS16 was tagged with mRFP at its C-terminus to produce the vector Pro35S: NtUXS16-mRFP (Fig. 3A), and then the vector was transformed into BY-2 cells. The membrane and soluble fraction were prepared from non-transformed and transformed BY-2 cells by ultracentrifugation. After resolution of the proteins of sodium dodecyl sulfate (SDS)-polyacylamide gel electrophoresis (PAGE), only proteins in the membrane fraction from transgenic cell gave a fluorescent band at 68 kDa and another band (Fig. 3A), which suggested that NtUXS-mRFP was recovered in membrane fraction and might form a complex with other intact protein. Confocal microscopic observations of the fusion of NtUXS16 and mRFP (NtUXS16-mRFP) indicated that the fusion protein was localized to bright punctate structures in log-phase cells (Fig. 3B). Such red fluorescence was not observed in non-transformed cells (Fig. 3B). Weak fluorescence was sometimes observed in the vacuoles (Fig. 3B), suggesting that some of the NtUXS16-mRFP was targeted to the vacuoles for degradation. These observations indicated that NtUXS16-mRFP was localized in small organelles (e.g., Golgi apparatus) but not in larger organelles (e.g., mitochondria and/or plastids).

**Fig. 4:**
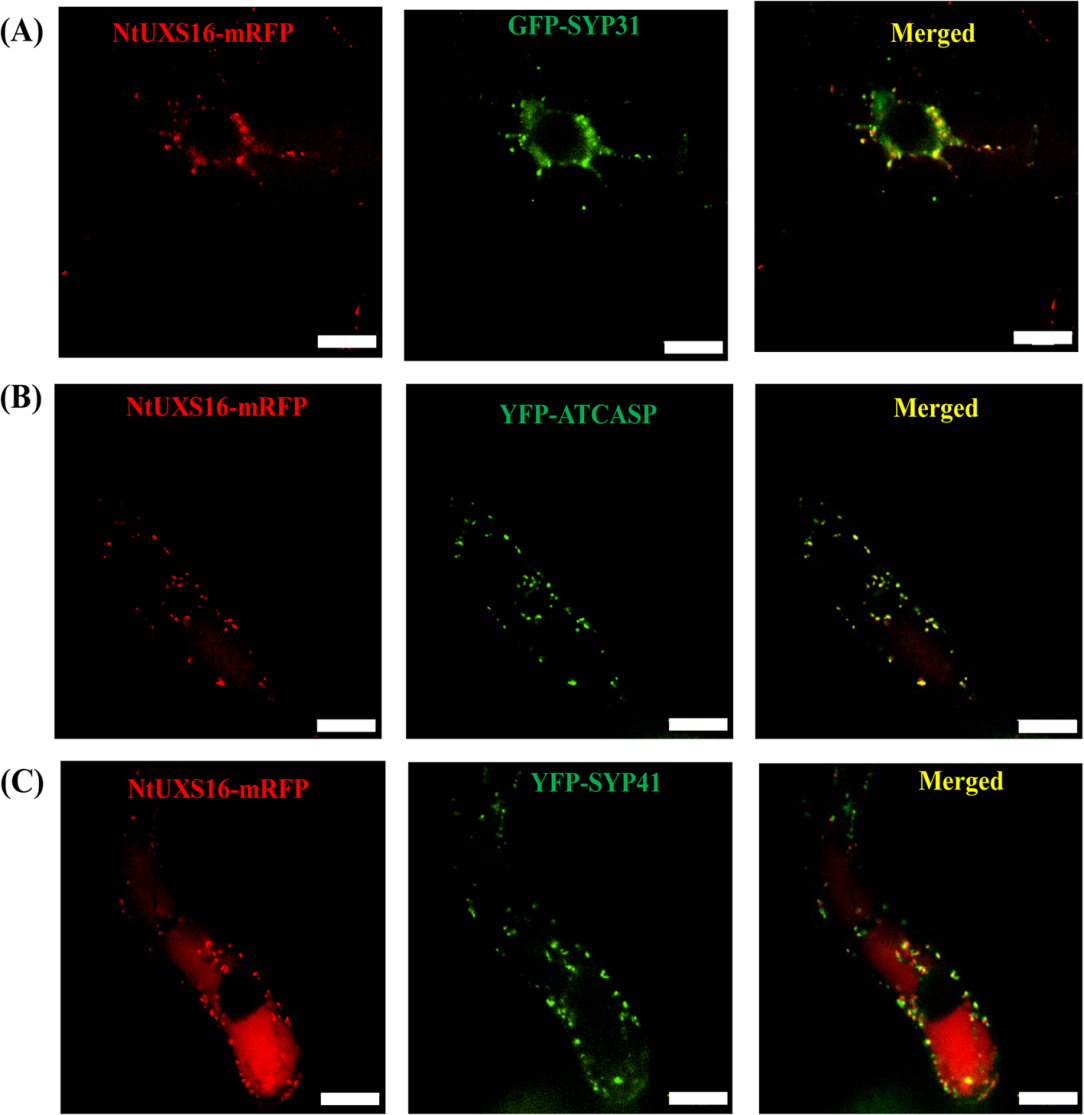
Subcellular localization of NtUXS16-mRFP with organelle markers. Localization of NtUXS16-mRFP with GFP-SYP3 (**A**), NtUXS16-mRFP with YFP-ATCASP (**B**), NtUXS16-mRFP with YFP-SYP41 (**C**). Bars = 10 μm. The right most number is the percentage of the co-localized green and red dots. Bars = 10 µm, n ≥ 10 cells

To assess the localization of NtUXS16-mRFP, we examined the transgenic cell lines expressing NtUXS16-mRFP and Golgi markers, including the cis-Golgi marker GFP-SYP31, the medial-Golgi marker YFP-ATCASP and the trans-Golgi network/TGN marker YFP-SYP41 [28–30]. GFP-SYP31 and NtUXS16-mRFP displayed fluorescence patterns that closely resembled those of YFP-SYP41 and NtUXS16-mRFP. These proteins were positioned adjacent to each other, with some regions showing partial overlap (Fig. 4). In comparison to SYP41 and SYP31, the majority of the NtUXS16-mRFP positive dots displayed a positive YFP-ATCASP signal. This observation suggested that both NtUXS16 and YFP-ATCASP were present in the same compartment, indicating their co-localization. Hence, it can be concluded that NtUXS16-mRFP was specifically localized in the medial-Golgi apparatus.

### Effects of BFA on NtUXS16-mRFP in BY-2 cells

**Fig. 5:**
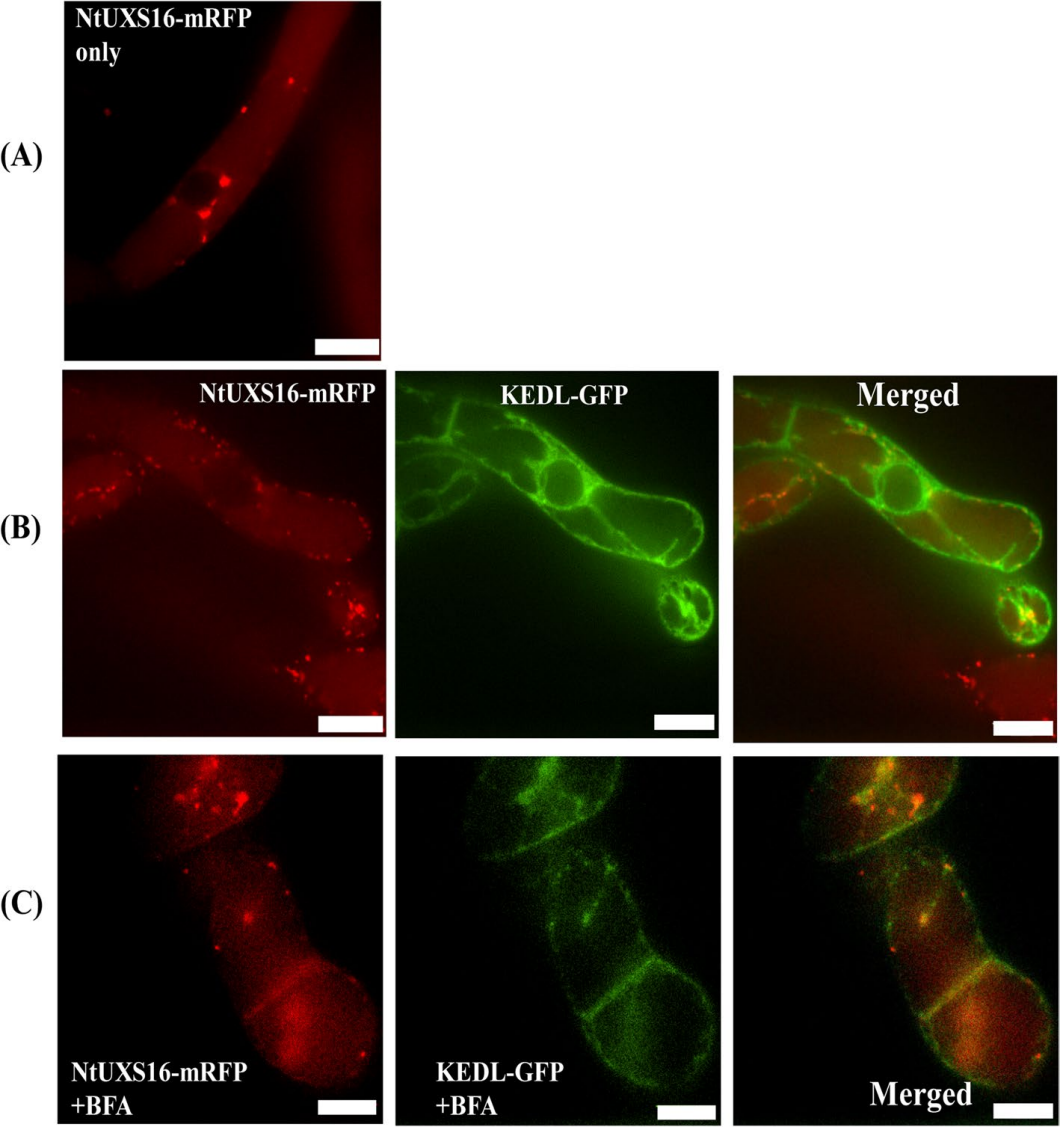
Subcellular localization of NtUXS16 in the presence of BFA. Effects of BFA. BY-2 cells expressing NtUXS16-mRFP only (**A**), NtUXS16-mRFP and KEDL-GFP (**B**), NtUXS16-mRFP and KEDL-GFP was treated with 5 μg/mL of BFA for 120 to 140 min (**C**), bars = 10 µm, n ≥ 10 cells

BFA inhibits protein trafficking in the secretory pathway by inhibiting vesicle formation and disrupting the cis, medial, and trans cisternae of the Golgi apparatus. KDEL-GFP is a green-fluorescent marker for the endoplasmic reticulum (ER). We investigated the transgenic cell line that expressed NtUXS16-mRFP and KDEL-GFP simultaneously (Fig. 5A). As depicted in Fig. 4, it was observed that the majority of mRFP punctates did not overlap with the green fluorescence, suggesting that NtUXS16-mRFP was absent in certain areas of the ER. In presence of BFA, the NtUXS16-mRFP localization was redistributed in the BY-2 cells (Fig. 5C). Compared with the localization of KEDL-GFP in presence of BFA (Fig. 5B), NtUXS16-mRFP punctate fluorescence signals were perinuclear signals, where GFP fluorescence was detected (Fig. 5C). All these results confirmed that NtUXS16-mRFP is localized in the Golgi apparatus.

### NtUXS16 is recovered in microsomal membrane fraction

**Fig. 6:**
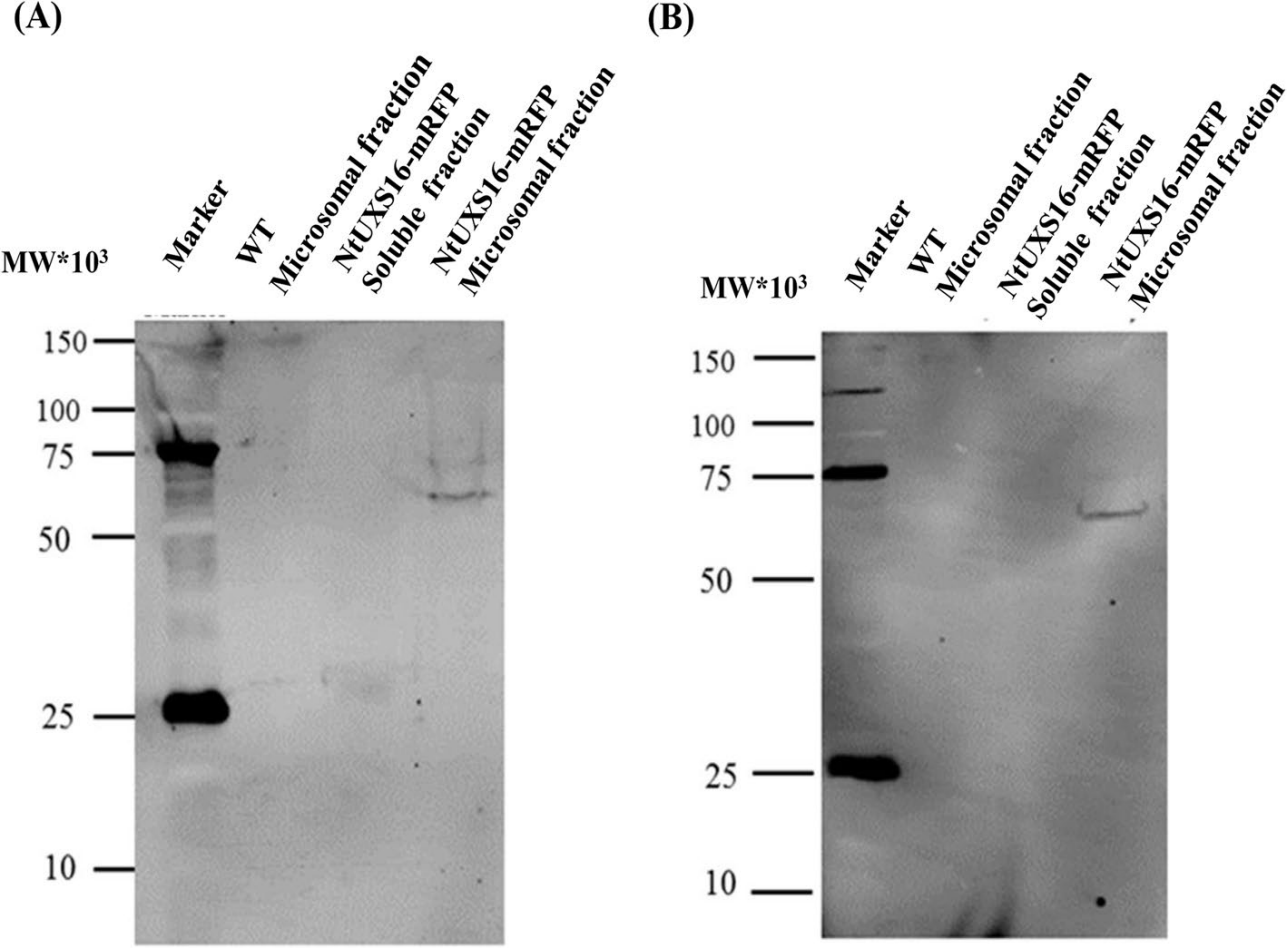
Immunoblot analyses of NtUXS16 in microsomal proteins from non-transformed and of NtUXS16-mRFP-expressing BY-2 cells. Notes: Equal amounts of microsomal proteins from non-transformed BY-2 cells (WT) or transformed BY-2 cells expressing NtUXS16-mRFP were separated by SDS-PAGE, transferred to PVDF membranes and probed with anti-RFP antibody (**A**) or antibodies of NtUXS16 (**B**)

To verify the integrity of the NtUXS16-mRFP fusion protein localized to the Golgi, as well as to investigate the association of this protein and endogenous NtUXS16 with the membrane, we conducted an immunoblot analysis. This analysis involved the use of anti-NtUXS16 antibody against the microsomal fraction obtained from both wild-type and transformed BY-2 cells. Anti-NtUXS16 antibody was generated using the synthetic peptide CYTPKPRKPWQNVIRP, which corresponds to the 20th to 33rd amino acids of NtUXS16. This sequence is located prior to the predicted transmembrane region and was employed as an antigen through the incorporation of a cysteine for KLH conjugation (Fig. 6A). Immunoblot analysis was conducted against microsomal proteins from non-transformed BY-2 cells with purified anti-NtUXS16 antibody. Microsomes from transgenic tobacco BY-2 cells expressing NtUXS16-mRFP generated a single band, which migrated at approximately 68 kDa. The size of the polypeptides roughly matches the size of fusion protein NtUXS16-mRFP (Fig. 6A). And no band was detected from the soluble fraction of the transgenic cell line using the anti-NtUXS16 antibody (Fig. 6A). The 68 kDa polypeptide was also detected by the anti-RFP antibody in microsomal proteins from NtUXS16-mRFP-expressing cells (Fig. 6B). All the data indicated that NtUXS16-mRFP associated with membranous organelles is an intact form and suggested that the observed signals accurately represent its distribution.

### Topology to membrane of NtUXS16 protein

**Fig. 7:**
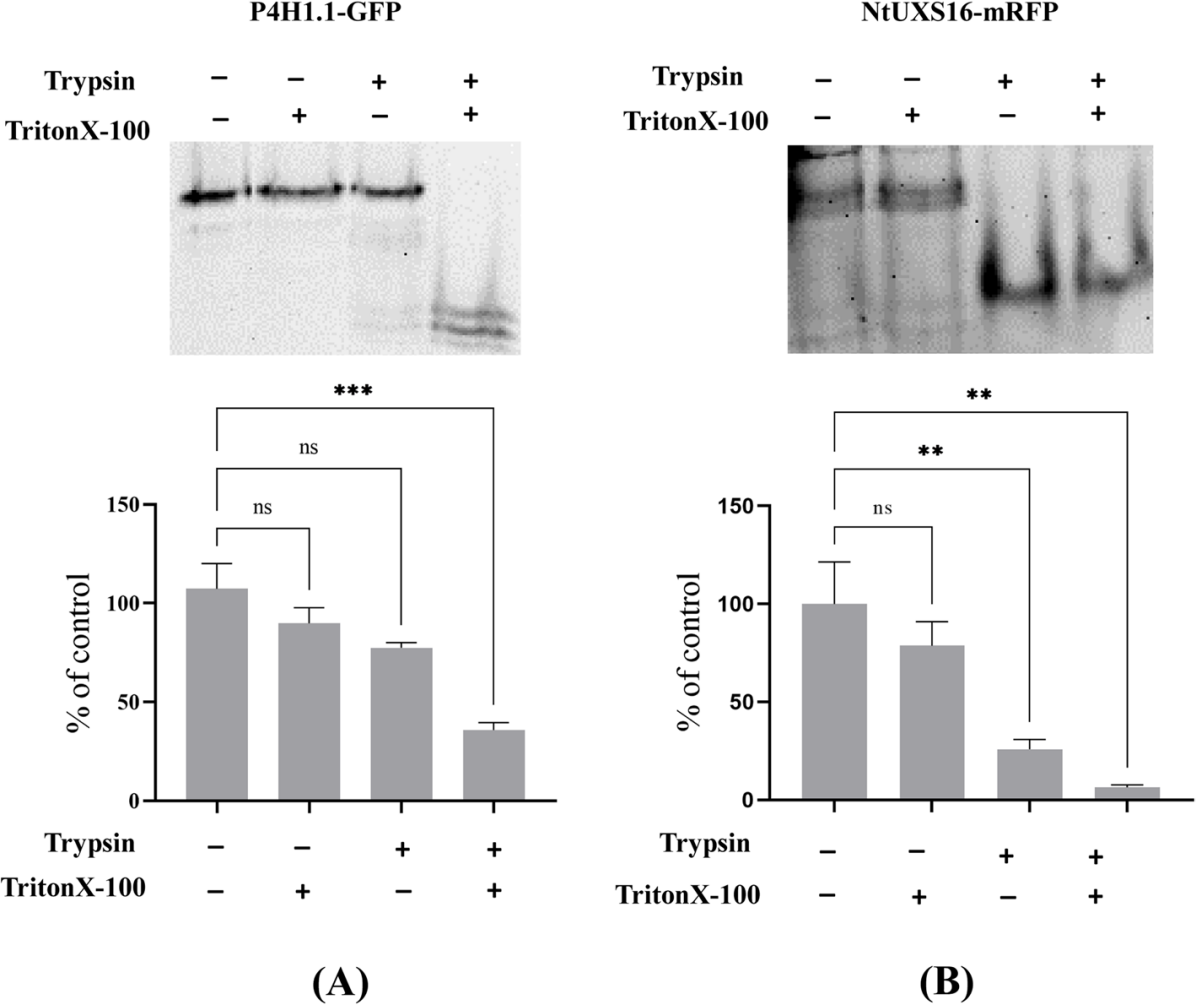
NtUXS16 is not a type II membrane protein. **A** Protease resistance of tobacco NtUXS16. Microsomes from transformed BY-2 cells expressing either NtUXS16-mRFP, NtP4H1.1-GFP were treated at 37 °C for 1 h with trypsin in the presence or absence of Triton X-100. Subsequently, proteins were separated by SDS-PAGE and the fluorescence of GFP or mRFP fusion proteins were detected as described in Materials and Methods. **B** Quantitative results are shown for the intact form of each protein in (A). The percentages of the intensity of signals relative to no trypsin and no Triton X-100 signals are shown. Experiments were repeated 3 times and gave basically identical results. Data were presented as mean ± standard deviation (s.d.) and analyzed using Student’s *t*-test (n ≥ 30; **, *P* < 0.01)

To determine the NtUXS16 orientation of the C-terminal domain relative to the membrane, transgenic BY-2 cells expressing NtUXS16-mRFP were used to investigate the membrane topology by protease susceptibility. Microsomal fractions were treated with trypsin in the presence or absence of Triton X-100. Subsequently, the digested proteins were separated by SDS-PAGE and analyzed by immunoblotting as previously described [23]. In the absence of trypsin and Triton X-100, a fluorescent band of NtUXS16-mRFP was detected, while a protein band of similar intensity was also detected with Triton X-100 only (Fig. 7). After trypsin treatment, the intensity of the fusion protein decreased significantly and several smaller fluorescence bands appeared. In the presence of both Triton X-100 and trypsin, the intact fluorescent band almost completely disappeared (Fig. 7B). Two membrane proteins were used as control. NtP4H1.1 was a Golgi type II membrane protein, which was resistant to trypsin digestion in the absence of detergent. The other membrane protein was GLMT1 with the N-terminal region and the C-terminal catalytic region located on the cytosolic side of the Golgi apparatus [31]. The quantitative analysis of intact bands indicated NtUXS16-mRFP was more sensitive than that of NtP4H1.1-GFP (Fig. 7B). The sensitivity of NtUXS16-mRFP to trypsin was similar to that of GLMT1-mRFP, and the two fusion proteins were almost completely digested in the presence of Triton X-100 and trypsin (Fig. 7A) [23]. These results suggested that the catalytic domain of NtUXS16-mRFP was located on the cytosolic side of the Golgi apparatus, and suggested that this protein was not a type II membrane protein.

### Overexpression of *NtUXS16* increased the hypocotyl length in transgenic *Arabidopsis*

**Fig. 8:**
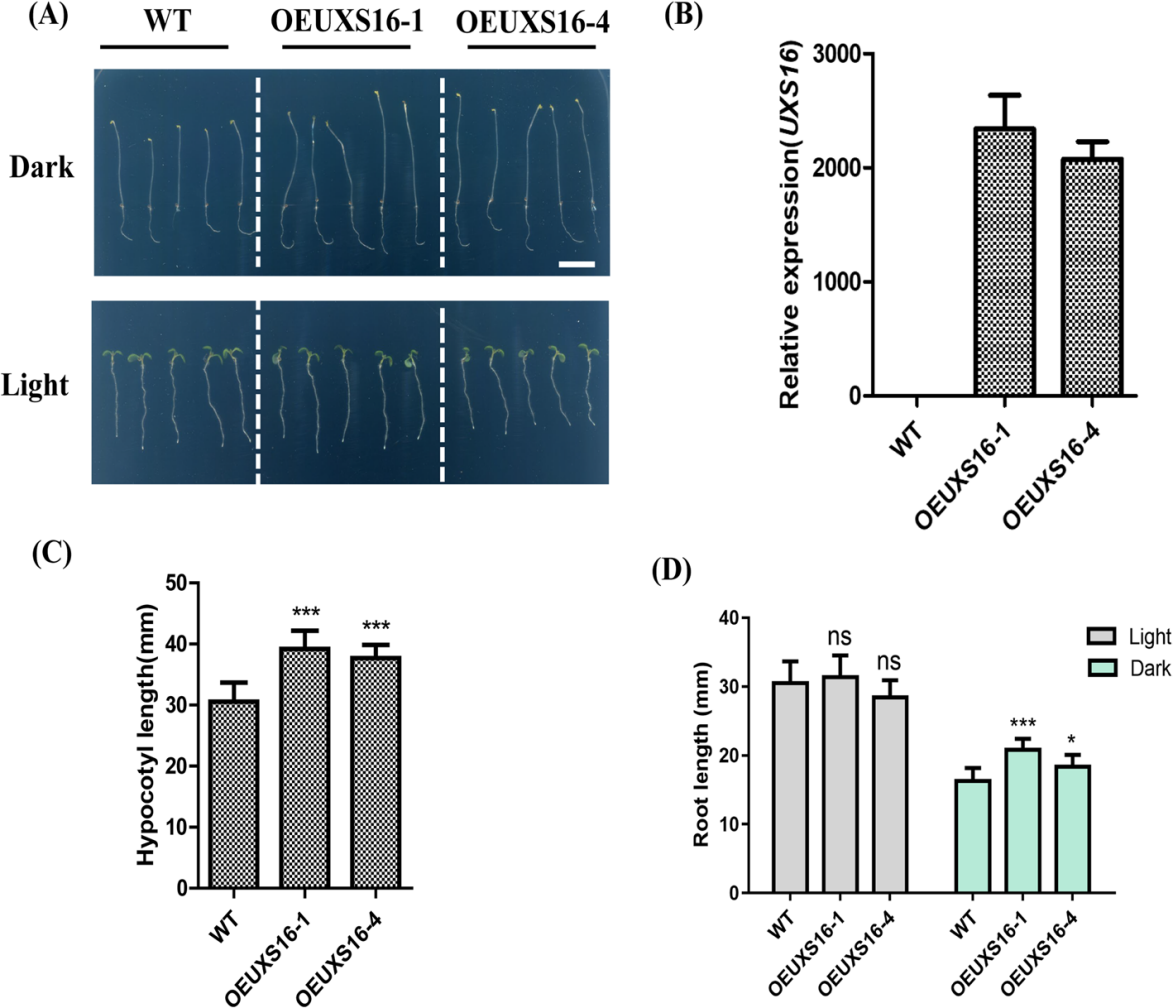
Hypocotyl phenotypes of dark/light-grown WT, OEUXS16-1 and OEUXS16-2. Overall plant morphologies of WT, OEUXS16-1 and OEUXS16-2 seedlings grown on 1/2 MS medium in the dark or under light (**A**). Bars = 1 mm. **B** Expression level analysis of *NtUXS16 *were detected by qPCR. Total cellular RNAs were extracted from the aerial parts of 4-week-old plants. **C** Average hypocotyl length of dark-grown WT, OEUXS16-1and OEUXS16-2 grown on 1/2 MS medium. Data were presented as mean ± standard deviation (s.d.) and analyzed using Student’s *t*-test (n ≥ 30; **, *P* < 0.01). **D** Average hypocotyl and root length of light-grown 7-day-old WT, OEUXS16-1 and OEUXS16-2 grown on 1/2 MS medium. Data were presented as mean ± s.d. and analyzed as in (**C**) (n ≥ 30; **, *P* < 0.01)

To further establish the function of NtUXS16, *NtUXS16* was ectopically expressed in Arabidopsis. The 35S::NtUXS16 vector was generated and then used to transform Arabidopsis. Two independent T_3_ lines homozygous for the transgene (OEUXS16-1 and OEUXS16-2) were selected for further analysis. Both OEUXS16-1 and OEUXS16-2 showed higher relative expression comparison with wild type (Fig. 8B). We measured the root and hypocotyl length of transgenic and WT seedlings under the dark and long-day conditions (16 h light/8 h dark). The hypocotyl lengths of transgenic seedlings were found to be longer than the WT at 5 DAG (days after germination) in dark, as shown in Fig. 8A and C. The growth of wild type roots was found to be significantly inhibited under dark conditions (Fig. 8D). However, in contrast, the transgenic seedlings exhibited longer root lengths compared to the wild type (Fig. 8D). These results showed that the NtUXS16 gene positively regulated hypocotyl-root elongation under the dark.

## Discussion

### Structural and evolutionary relationships of UXS gene family in tobacco

UDP-glucuronate decarboxylase (UXS) can irreversibly catalyze the biosynthesis of UDP-Xyl from UDP-glucuronic acid (UDP-GlcA) and plays a key role in xylan biosynthesis. Until now, only a limited number of UXS genes have been identified in plants, such as rice (6 genes) [14], Arabidopsis (6 genes) [11], and *Populus tomentosa* (7 genes) [32]. In this study, a total of 17 UXS genes were identified and characterized in tobacco. All 17 NtUXSs are hydrophilic proteins that contained a N-terminal ADP-binding sequence GxxGxxG related to NAD(P) binding and a conserved YxxxK motif. Based on phylogenetic analyses, the NtUXSs that were identified have been categorized into two distinct groups, namely Group I and Group II. Additionally, the UXSs in Group II can be further subcategorized into two subgroups, namely Group IIa and Group IIb. (Fig. 1). The conserved GxxGxxG was differentiated as GGAGFVG and GGAGFVG for Group I and II NtUXS proteins, respectively, which was identical to the results in Arabidopsis [11]. In addition, Ser, together with Tyr and Lys, which made up the catalytic triad in the YxxxK motif [10], were found to be the prevailing and highly conserved residues among NtUXSs (Figure S2). In addition, NtUXSs clustered together exhibited the similar conserved motif distribution, and the numbers of introns in NtUXS genes within Group I and II were 11 and 6, respectively (Fig. 2), indicating that the conservative motif and intron numbers of each group of NtUXSs are highly conserved.

### NtUXS16 is localized to the Golgi apparatus in tobacco BY-2 cells

The Arabidopsis thaliana genome contains a total of six UXS genes. Among these, UXS1, UXS2, and UXS4 encode Golgi-localized enzymes, while UXS3, UXS5, and UXS6 encode cytosol-localized enzymes [10, 11]. In tobacco, we analyzed the subcellular localization of NtUXS16 and found that the NtUXS16-mRFP was localized to the medial-Golgi apparatus (Fig. 4B). NtUXS16 exhibited the greatest homology with AtUXS2 and AtUXS4 in Arabidopsis (Fig. 1), indicating potential similarities in their functions. Our study involved protease digestion experiments using microsomes from NtUXS16-mRFP-expressing cells, which revealed a significant reduction in the intact form of the fusion protein upon exposure to trypsin. Several smaller fluorescence bands appeared, and the fluorescent band for the intact size almost completely disappeared in the presence of both Triton X-100 and trypsin. Therefore, this data suggested that the C-terminal of NtUXS16-mRFP is located on the cytosolic side of the Golgi apparatus, and NtUXS16 is probably not a type II membrane protein (Fig. 7). This discovery is different from the former prediction, which suggested that NtUXS16 might have specific functions in the Golgi apparatus.

### The new function about the Golgi apparatus in plants

The functions of Golgi apparatus are dependent on the property of enzymes and trafficking proteins. Different Golgi region contains different proteins. For example, in the carbohydrate synthesis stage, the main function of these resided enzymes is to catalyze the glycosylation of proteins and lipids, and the synthesis of polysaccharides [33–35]. The Golgi glycosylation enzymes have a type II topology with a cytosolic N-terminus facing the Golgi lumen and a transmembrane domain [36]. But in the carrier formation stage, some biosynthesis and processing also occurs. Type I TGN proteins catalyze proteolytic cleavage of prohormones in mammalian and fungal cells [37]. Therefore, the topology of proteins plays an essential role in the function of the Golgi apparatus. In plants, the Golgi apparatus is a factory for the biosynthesis of complex polysaccharides in the cell wall. NtUXSs convert UDP-GlcA to UDP-Xyl, which is an important sugar donor required for the synthesis of plant cell wall polysaccharides, plant metabolites and glycoprotein [4, 37, 38]. In tobacco, NtUXSs are also classified into two types: membrane proteins and cytoplasm protein-like homologs in others species [10, 11]. Some biochemical evidences suggested that most activities of NtUXSs were from the soluble fraction, just 10% of the activities were associated with the microsomal fraction [12]. Despite extensive research, the exact mechanism behind the presence of multiple isoforms in plants and the necessity for consistent enzymatic activities across various sub-compartments remains unclear. NtUXS16 was proved to localize to medial-Golgi (Fig. 3). Based on the former research works, UDP-xyl was proved to have the dual function: being a precursor for synthetizing glyco-conjugate or being a molecular sensor inhibiting the activity of cytosolic UDP-glucose dehydrogenase [6, 11]. Therefore, the function of NtUXS16 in Golgi apparatus needed to be investigated. In this study, NtUXS16 is probably not a type II membrane protein, and the C-terminal of NtUXS16-mRFP seems to localize on the cytosolic side of the Golgi apparatus (Fig. 7). These data suggested that NtUXS16 might have the same function as cytosolic NtUXSs, or NtUXS16 might have a novel function of transforming the UDP-xyl into Golgi lumen to synthetize the polysaccharides with the interact proteins.

### NtUXS16 gene positively regulated hypocotyl-root elongation response to light

The growth and development of plants are intricately influenced by a combination of photoreceptors, hormones, and transcription factors, all of which are modulated by light. Furthermore, light plays a significant role in the elongation of the hypocotyl in seedlings. In Arabidopsis, the expression patterns of the six UXS genes were discovered to overlap and diverge in young seedlings, stems, and hypocotyl-root [18]. The UXSs localized in the cytosol exhibited significantly higher enzymatic activity than that in the Golgi [18]. However, in our study, we have shown that the overexpression of NtUXS16 led to a notable elongation of the hypocotyl and root length in transgenic Arabidopsis seedlings in the absence of light, as compared to the wild-type. Hence, there is a potential for the NtUXS16 enzyme, responsible for converting UDP-GlcA to UDP-Xyl in cell wall biosynthesis, to be influenced by light and play a role in the elongation of hypocotyl-root in the absence of light.

### Supplementary Information

**Additional file 1: Supplementary Figure 1.** Transmembrane prediction of NtUXS proteins by TMHMM server version 2.0. **Supplementary Figure 2.** Alignment of amino acid sequences of NtUXS proteins. The conserved motifs GxxGxxG and YxxxK are boxed with red and green, respectively. The catalytic Ser residue was marked as triangle. **Supplementary Table S1.** Primers used in this study. **Supplementary Table S2.** The accession numbers of *UXS* family genes used in this study. **Supplementary Table S3.** Sequences and lengths of motifs among the *UXS* gene family members in *Nicotiana tabacum*.

**Additional file 2: Table S4. **The sequence of *UXS *gene family in *Nicotiana tabacum.*

## Data Availability

The datasets generated and/or analyzed during the current study are available in the following open access repositories. Accession numbers of all datasets and the CDS and protein sequences of tabacco are also provided in the supplementary information tables. Rice Genome Hub [https://rice-genome-hub.southgreen.fr/]. RiceXPro [https://ricexpro.dna.affrc.go.jp/]. TAIR[https://www.arabidopsis.org/download/index-auto.jsp?dir=%2Fdownload_files%2FGenes%2FAraport11_genome_release].
