# Supplementary material for: Comprehensive analysis of the UDP-glucuronate decarboxylase (UXS) gene family in tobacco and functional characterization of NtUXS16 in Golgi apparatus in Arabidopsis
Source: BMC Plant Biol. 2023 Nov 8;23:551. doi: 10.1186/s12870-023-04575-3 (PMC10631120; doi:10.1186/s12870-023-04575-3)
Supplement: Supplementary file 1 — Additional file 1: Supplementary Figure 1. Transmembrane prediction of NtUXS proteins by TMHMM server version 2.0. Supplementary Figure 2. Alignment of amino acid sequences of NtUXS proteins. The conserved motifs GxxGxxG and YxxxK are boxed with red and green, respectively. The catalytic Ser residue was marked as triangle. Supplementary Table S1. Primers used in this study. Supplementary Table S2. The accession numbers of UXS family genes used in this study. Supplementary Table S3. Sequences and lengths of motifs among the UXS gene family members in Nicotiana tabacum. [file 12870_2023_4575_MOESM1_ESM.pdf]

Supplementary Material

Comprehensive Analysis of the UDP-Glucuronate Decarboxylase (UXS) Gene Family in Tobacco and Functional Characterization of *NtUXS16* in Golgi apparatus in Arabidopsis

Zhimin Li [1,#], Runping Chen [2,#], Yufang Wen [2], Hanxiang Liu[2], Yangyang Chen[2], Xiaoyu Wu [1], Youxin Yang[3], Xinru Wu[4], Yong Zhou [1,5]* and Jianping Liu[1]*

*** Correspondence:** Jianping Liu: JianpingLiu@jxau.edu.cn

1 Supplementary Data

2 Supplementary Figures and Tables

2.1 Supplementary Figures

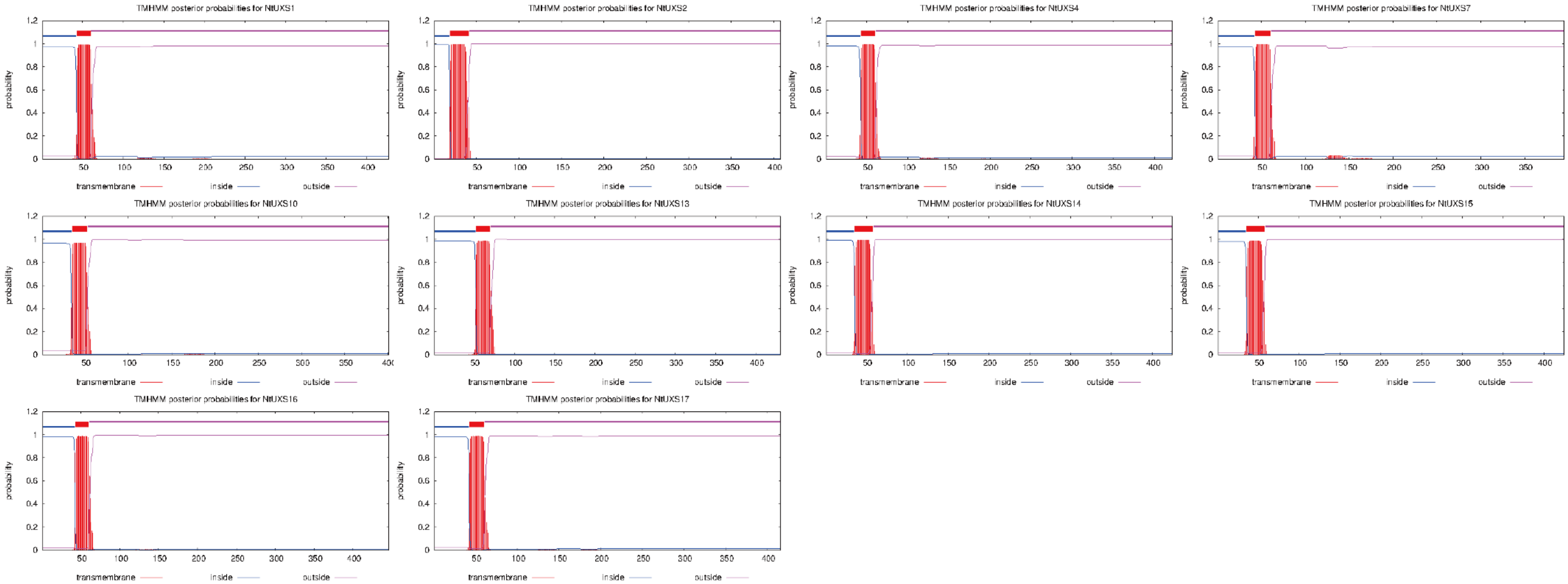

Supplementary Figure 1. Transmembrane prediction of NtUXS proteins by TMHMM server version 2.0.

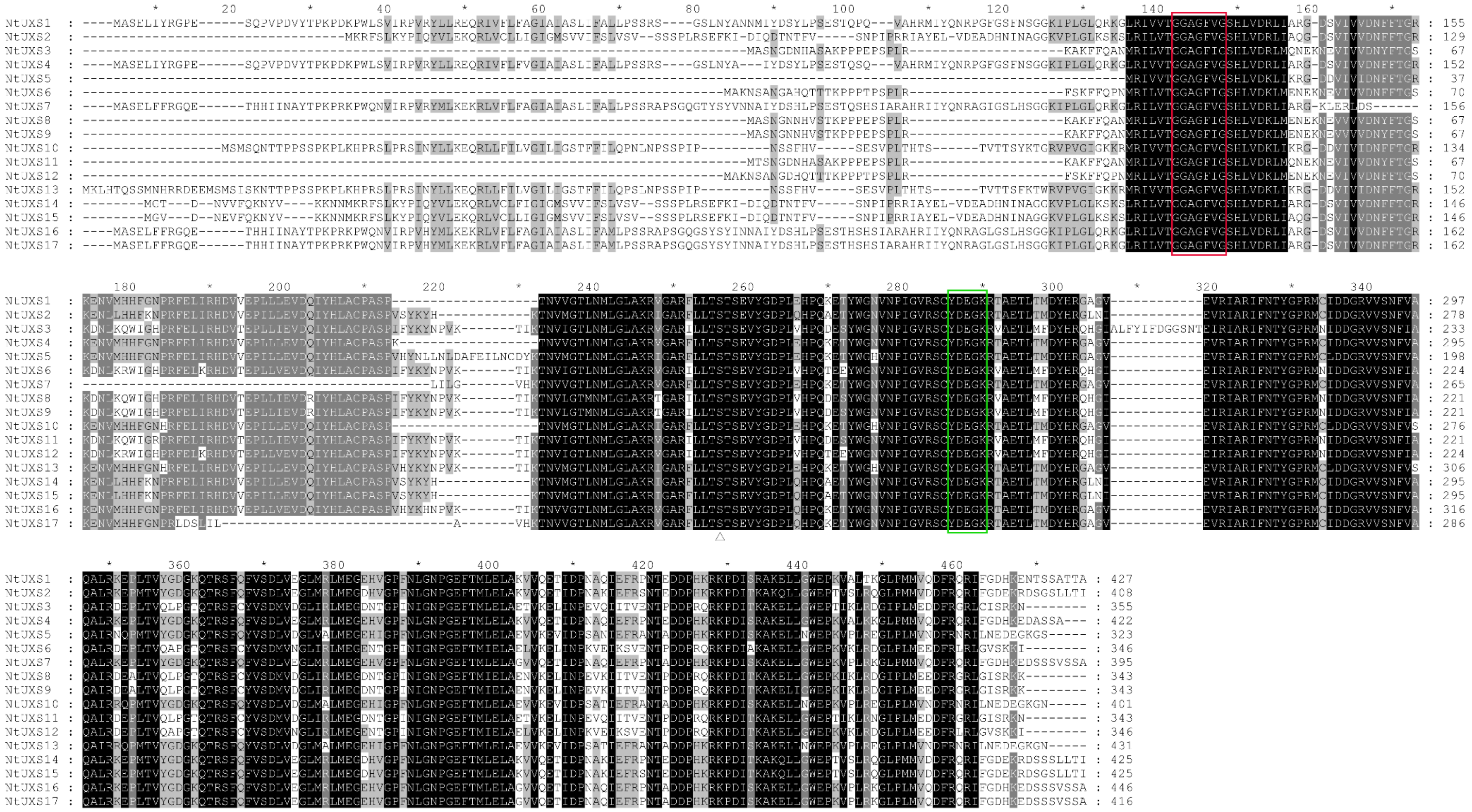

Supplementary Figure 2. Alignment of amino acid sequences of NtUXS proteins.The conserved motifs GxxGxxG and YxxxK are boxed with red and green, respectively. The catalytic Ser residue was marked as triangle.

2.2 Supplementary Tables

Supplementary Table S1. Primers used in this study.

**Gene**	**Primer name**	**Sequences**	**Use**
NtUXS16	NtUXS16qPCRf	GCATAACCCCGTCAAGACCA	qRT-PCR
NtUXS16	NtUXS16qPCRr	TAAACTTCGCTGGTGCTCGT	qRT-PCR
Actin8	Actin8qrtF	GCCGATGCTGATGACATTC	qRT-PCR
Actin8	Actin8qrtR	CTCCAGCGAATCCAGCCTTA	qRT-PCR
NtUXS16	NtUXS16 -mRFPF	AtgcAAGCTTATGGCTTCTGAATTGTTC	mRFP fusion
NtUXS16	NtUXS16 -mRFPR	GCATTCTAGACGCGGAGGAGACAG	mRFP fusion
NtUXS16	OE NtUXS16	AtgcCTGCAGATGGCTTCTGAATTGTTC	Overexpression
NtUXS16	OE NtUXS16	GCATTCTAGACGCGGAGGAGACAG	Overexpression

Supplementary Table S2. The accession numbers of *UXS* family genes used in this study.

Species	Name	Gene ID
Nicotiana tabacum	NtUXS1	Ntab0068330
	NtUXS2	Ntab0461190
	NtUXS3	Ntab0221920
	NtUXS4	Ntab0289010
	NtUXS5	Ntab0831500
	NtUXS6	Ntab0946790
	NtUXS7	Ntab0362020
	NtUXS8	Ntab0368380
	NtUXS9	Ntab0751250
	NtUXS10	Ntab0941870

	NtUXS11	Ntab0259700
	NtUXS12	Ntab0219810
	NtUXS13	Ntab0603520
	NtUXS14	Ntab0600090
	NtUXS15	Ntab0038020
	NtUXS16	Ntab0485970
	NtUXS17	Ntab0561250
Arabidopsis thaliana	AtUXS1	AT3G53520.4
	AtUXS2	AT3G62830.1
	AtUXS3	AT5G59290.2
	AtUXS4	AT2G47650.2
	AtUXS5	AT3G46440.1

	AtUXS6	AT2G28760.2
Populus tomentosa	PtUXS1	KC311162
	PtUXS2	KC311163
	PtUXS3	KC311164
	PtUXS4	KC311165
	PtUXS5	KC311166
	PtUXS6	KC311167
	PtUXS7	KC311168
Oryza sativa	OsUXS1	AB167396
	OsUXS1	AB167397
	OsUXS3	AB079064
	OsUXS4	AB079063
	OsUXS5	AB182636
	OsUXS6	AB183706

Supplementary Table S3. Sequences and lengths of motifs among the *UXS* gene family members in *Nicotiana tabacum*.

Motif	Conserved amino acid sequences	Width
1	LLTSTSEVYGDPLEHPQKETYWGNVNPIGVRSCYDEGKRTAETLTMDYHR	50
2	IARIFNTYGPRMCIDDGRVVSNFVAQAJRKEPLTVYGDGKQTRSFQYVSD	50
3	NPGEFTMLELAEVVKETIBPNAQIEFRPNTPDDPHKRKPDISKAKELLGW	50
4	IVVDNFFTGRKENLMHHFGNPRFELIRHDVVEPJLLEVDQIYHLACPASP	50
5	KNLRILVTGGAGFVGSHLVDRLIARGDSV	29
6	EPKVPLRDGLPLMVDDFRQRJ	21
7	KTNVMGTLNMLGLAKRVGARF	21
8	KPWFSLIRPIRYLLKEQRLVFLFAGIAIASLIFALLPSSRSPSRSEFKSY	50
9	GLMRLMEGEHVGPFN	15
10	NHLTTKPPPEPSPLRKAKFFQ	21
